# Revision of an Eight-lead Boston Scientific® Spinal Cord Stimulator With Epidural Migration: A Case Report

**DOI:** 10.7759/cureus.97316

**Published:** 2025-11-20

**Authors:** Nwadi Igwe, Anna Green, Seung J Lee, Kanchana Gattu, Thelma Wright

**Affiliations:** 1 Pain Management, University of Maryland Medical Center, Baltimore, USA; 2 Anesthesiology, University of Maryland Baltimore County, Baltimore, USA

**Keywords:** chronic pain management, clinical case report, lead complication, pain relief, post operative pain relief, spinal cord stimulation (scs), spinal neuromodulation

## Abstract

Spinal cord stimulation (SCS) is a minimally invasive neuromodulation technique utilized to manage chronic pain conditions such as complex regional pain syndrome (CRPS), post-laminectomy syndrome, and peripheral neuropathy. Despite its efficacy, complications, including lead migration, discomfort, and device malfunction, can occur. We present the case of a male patient in his 60s with a history of chronic left lower extremity pain who underwent SCS implantation. Initial management included a successful trial period followed by permanent implantation. However, postoperative imaging revealed lead migration from the T10-T12 to the T12-L1 vertebral bodies. A subsequent lead revision was performed, yet the patient later experienced new abdominal pain due to further lead migration, necessitating referral to neurosurgery for paddle lead placement. The revision procedure involved reopening the initial incision, carefully removing scar tissue, and repositioning the lead within the epidural space using fluoroscopic guidance. The lead was anchored with a self-anchoring mechanism, and the incision was closed meticulously. Post revision, the patient experienced significant pain relief, confirmed by stable lead positioning on imaging. Unfortunately, lead migration recurred months later, leading to the decision for paddle-lead implantation. This case highlights the complexities of SCS implantation, particularly lead migration, and the importance of meticulous surgical technique and preoperative planning. The challenges of scar tissue formation and lead maneuverability are discussed, with a focus on improving fixation mechanisms and exploring alternative lead designs. Lead migration is a significant complication in SCS implantation that can compromise pain management efficacy. Vigilant postoperative monitoring, prompt revision, and advancements in lead design and fixation are essential to optimizing patient outcomes and minimizing the need for further interventions

## Introduction

Spinal cord stimulation (SCS) is a well-established, minimally invasive neuromodulation technique used to manage chronic pain conditions. It is particularly effective in treating complex regional pain syndrome (CRPS), post-laminectomy syndrome, and peripheral neuropathy [1,2]. SCS works by delivering electrical pulses to the spinal cord, which modulate pain signals before they reach the brain, thus providing pain relief. The SCS system typically consists of electrodes implanted near the spinal cord and a pulse generator implanted elsewhere in the body [3,4]. Electrodes for the spinal cord stimulator can be implanted percutaneously with the cylindrical leads, and the paddle leads can be placed via a laminotomy [2-5]. Before permanent implantation, patients usually undergo a trial period of three to seven days with temporary cylindrical leads to assess the effectiveness of pain relief. Successful trials lead to the permanent implantation of the system, which includes an implantable generator.

Despite its effectiveness, SCS implantation carries a significant risk of complications, with an incidence rate of 30-40%. Common complications include lead migration, infection, discomfort, lead fracture, implantable pulse generator (IPG) malfunction, and seroma formation [2,6,7]. Lead migration poses a substantial challenge as it can significantly reduce the efficacy of pain management and often necessitates revision surgery.

This report describes the case of a patient undergoing treatment for chronic neuropathic pain who developed forceful retching during SCS implantation, resulting in excessive movement and subsequent device migration. We outline the resulting need for SCS revision and discuss implications for future techniques and optimization of perioperative management to reduce similar complications.

## Case presentation

A male patient in his 60s with a history of chronic left lower extremity foot and ankle pain who had undergone several foot and ankle surgical procedures presented to our clinic for further evaluation. During the initial presentation, he complained of severe, constant aching and throbbing pain in his left lower extremity with increased temperature sensitivity and allodynia. The patient had tried opioids and gabapentin but had discontinued using them due to GI side effects that he experienced. He had also tried acupuncture, physical therapy, and a Tens unit, which gave him minimal pain relief.

On physical examination, surgical scars were seen on his left foot and ankle. There was also decreased hair growth on the dorsal aspect of his left foot without color changes, and the left foot was exquisitely sensitive to touch. We also noted sensory deficits along the dorsal and plantar aspects of his left foot. The patient was managed with amitriptyline at night and as-needed acetaminophen for pain relief, and he was scheduled for a lumbar sympathetic block in coordination with physical therapy sessions. He underwent a series of lumbar sympathetic blocks, which he coordinated with his physical therapy sessions. On follow-up visits, the patient noted that he was getting good pain relief with the lumbar sympathetic blocks, but they were short-lived. He noted that the longest lasted approximately four weeks.

**Figure 1:**
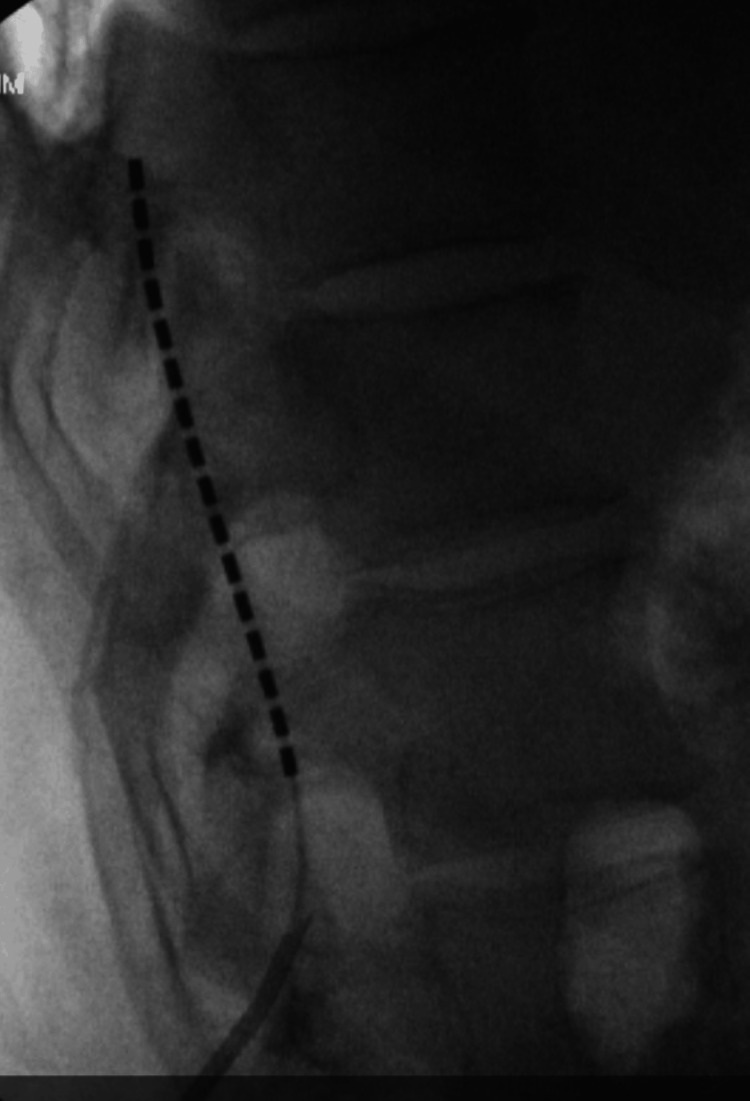
Lateral fluoroscopic image from trial placement

We discussed the placement of a spinal cord stimulator with the patient and his wife, and they were amenable to getting the spinal cord stimulator trial. The patient was cleared by pain psychology for the SCS trial, and he was scheduled for the procedure. The patient underwent an uneventful placement of a left-sided single 16-point lead (Boston Scientific Corporation, Marlborough, Massachusetts, United States) covering the T10-T12 vertebral bodies (Figure 1).

The trial was done over five days, and the patient got about 70% pain relief during the trial with improved quality of life and ability to complete ADLs. He was eager to go ahead with the permanent implantation. The patient was scheduled for implantation about two weeks after the trial. The procedure was scheduled in the main operating room, and the anesthetic plan was to utilize monitored anesthesia care.

**Figure 2:**
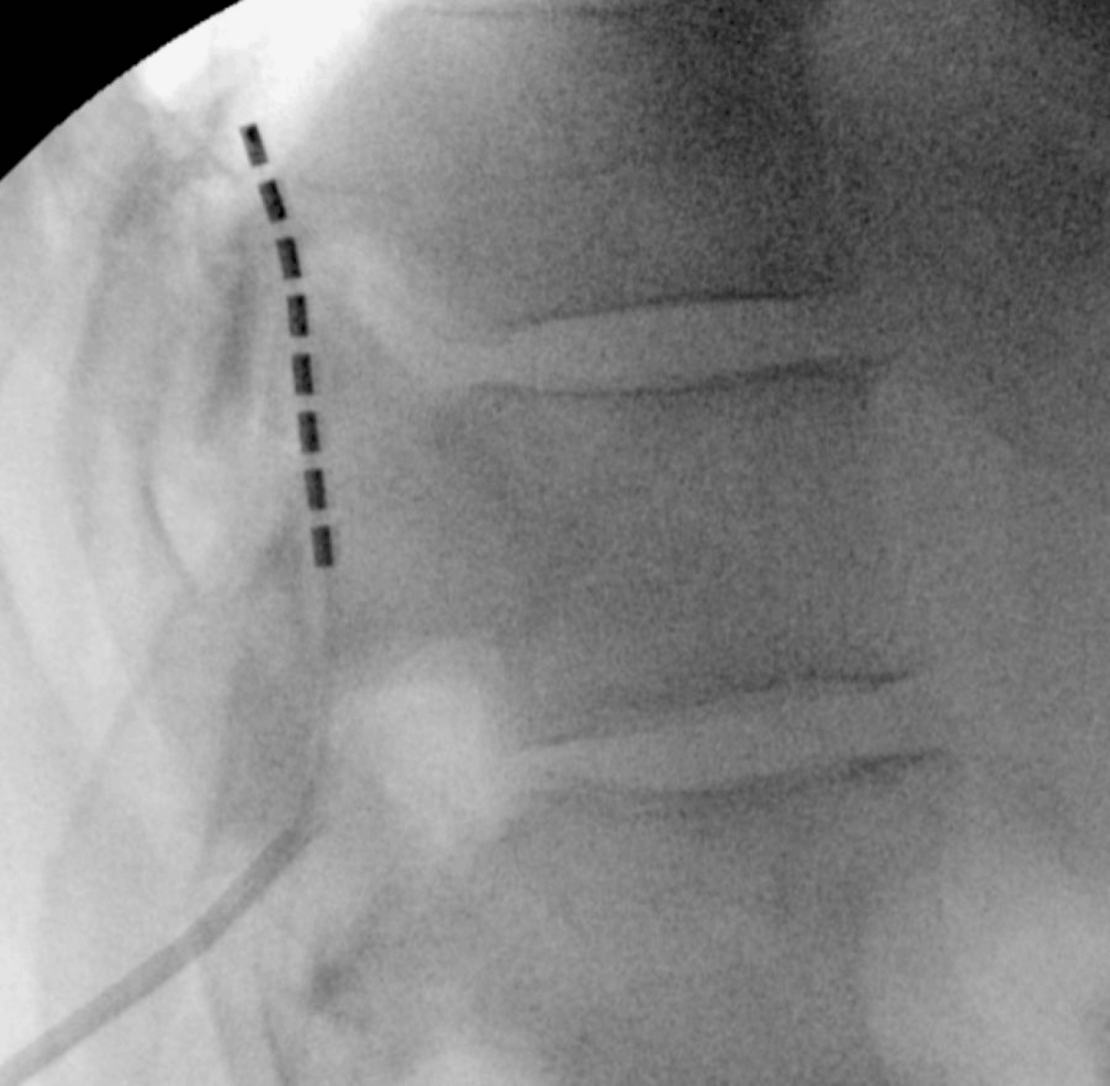
Lateral fluoroscopic image of permanent lead placement

The patient was placed in the prone position for the procedure and received 2 grams of cefazolin for prophylaxis. We initially attempted to access the epidural space using a straight 14-gauge Tuohy needle. However, after lateral imaging of the lead was performed, it was discovered to be located in the anterior epidural space. Several attempts were made to reposition the lead, which failed. We then decided to use a 14-gauge curved Tuohy needle, and an 8-contact lead (Boston Scientific Corporation) was placed at the T10 vertebral body (Figure 2).

**Figure 3:**
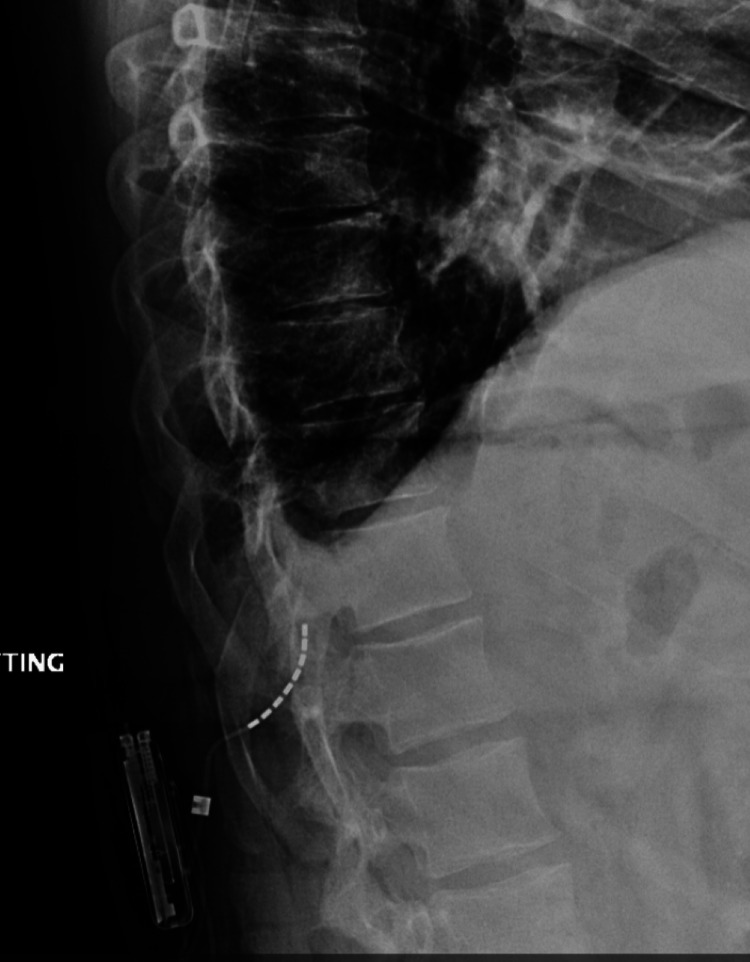
Thoracolumbar X-ray (lateral) showing lead migration

**Figure 4:**
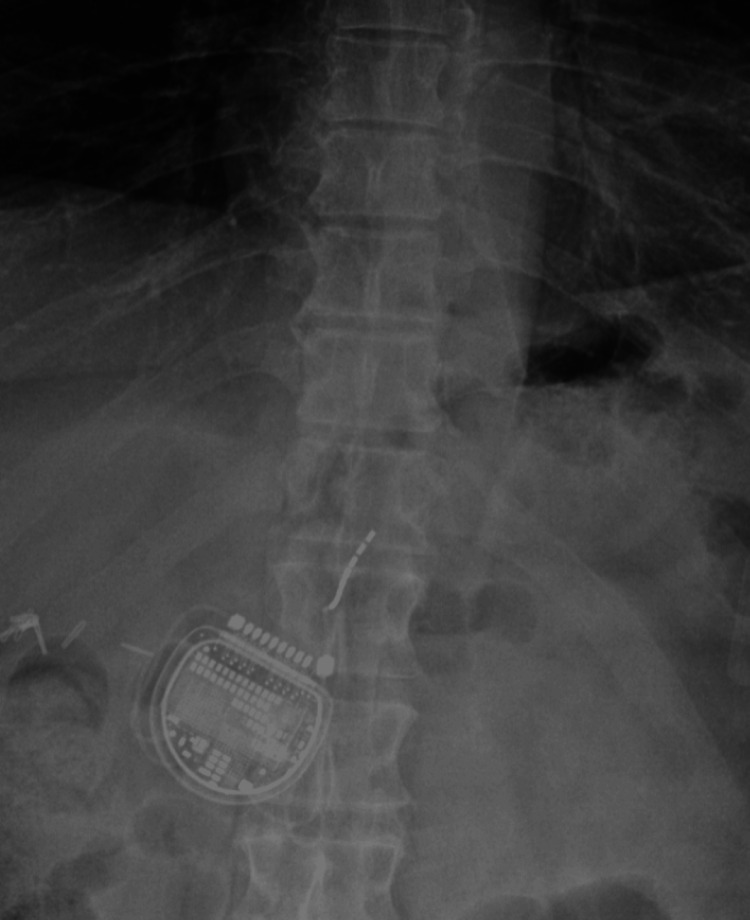
Thoracolumbar imaging (anteroposterior) showing the migrated lead

We then performed a vertical incision, and the IPG pocket was created lateral to the spinal column. Before anchoring the lead, the patient had a profound episode of retching and emesis. His oxygen saturation also decreased precipitously. Because incisions had been made and the IPG pocket created, we anchored and connected the lead to the battery. The patient was kept in the prone position throughout, and his airway was suctioned. His infusion of dexmedetomidine and remifentanil was stopped, and he was placed on 100% oxygen via face mask, which led to an improvement in his oxygen saturation. We then proceeded to close the pocket. The patient was transferred to the post-anesthesia care unit (PACU), and a postoperative thoracolumbar X-ray was ordered (Figures 3, 4). Upon review of the imaging, it was discovered that the lead had migrated out of the epidural space into the subcutaneous tissue. It now covered the T12-L1 vertebral bodies, not the T10-T12 as it was in the final fluoroscopic imaging.

The patient and his wife were informed of the findings. After discussing options with the patient, we decided on a lead revision as soon as possible. We scheduled the patient for a lead revision five days after the implantation due to OR availability. He was scheduled to undergo this procedure under general anesthesia due to the emesis at the last procedure.

**Figure 5:**
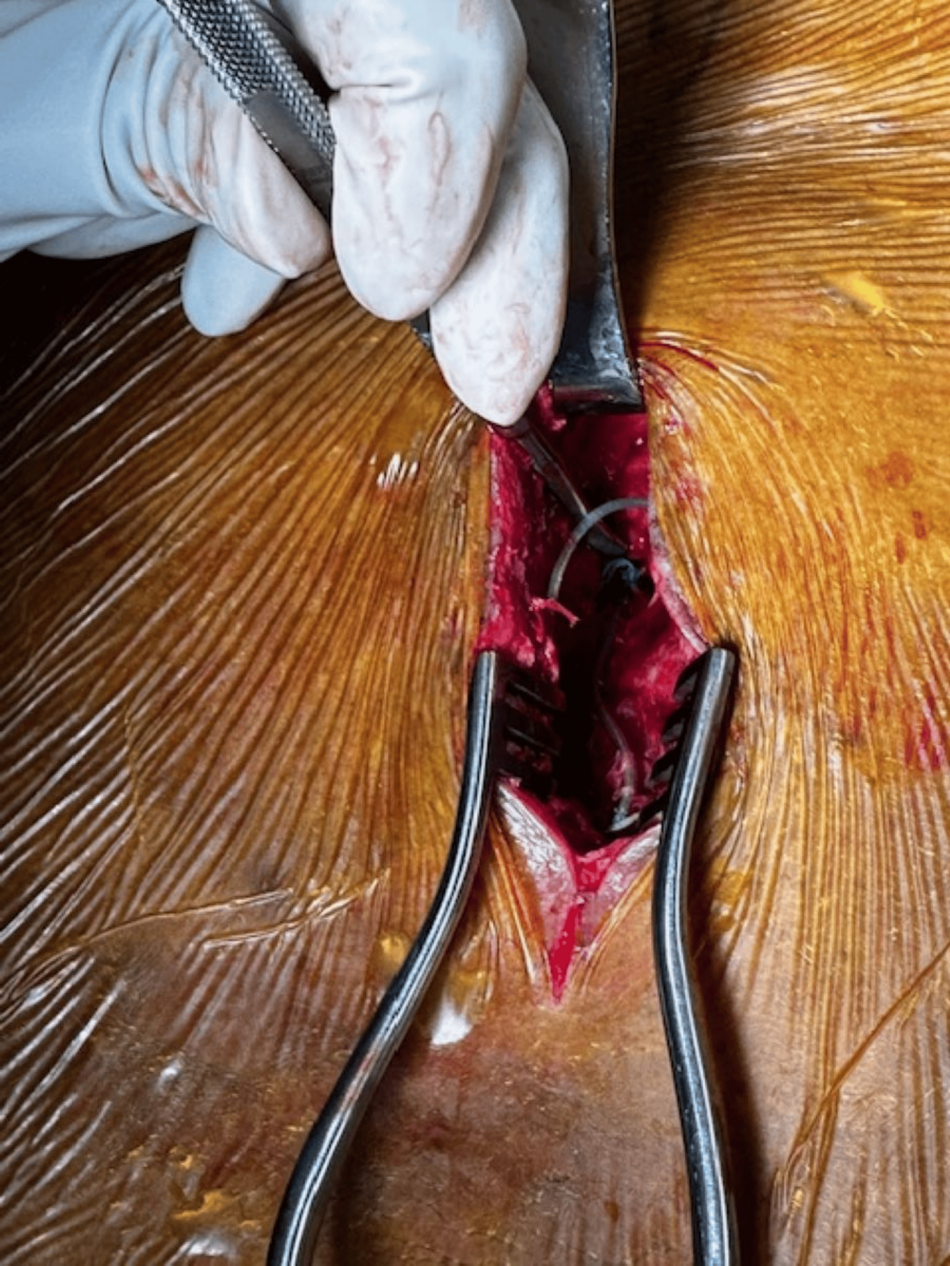
Image showing lead with anchor and some scar tissue formation

The back was draped, and sterile technique was strictly observed throughout the procedure. The old incision was reopened, and the existing stitches were removed. We noted that some parts of the lead already had some scar tissue (Figure 5).

Our attention was then turned to the battery, and it was removed carefully from its pocket. The hexagonal screw was then used to loosen up the end of the lead attached to the battery. After the screw was loosened, the lead was removed. We then carefully dissected out the rest of the lead. Initially, we were unable to find the anchor due to the formation of scar tissue around it. Using fluoroscopic guidance, we located the Clik anchor (Boston Scientific Corporation). We carefully dissected it, exposing the screw and two eyelets.

**Figure 6:**
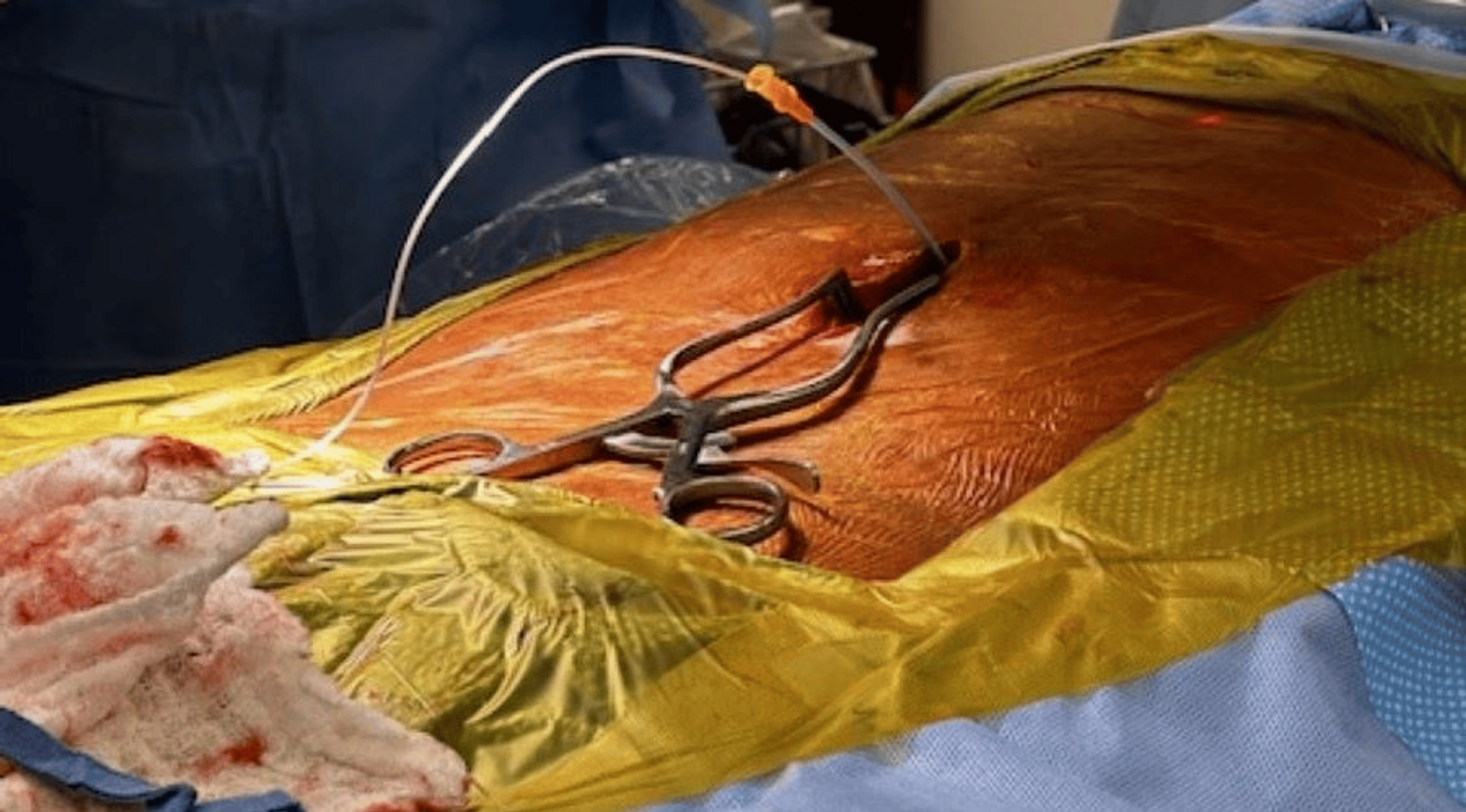
Image showing the introducer placed over the lead which was used to advance the lead further into the epidural space

The long hexagonal screw was used to loosen the grip on the lead. After this was done, a curved stylet was passed through the lead to the tip of the lead. We tried to guide the lead further into the epidural space, but were unable to do so with the curved stylet, so we tried using the straight stylet. Due to our inability to advance the lead with the stylet, we detached the anchor from the fascia and removed it from the lead to pass an introducer over the lead to aid in guiding the lead into the epidural space (Figure 6).

**Figure 7:**
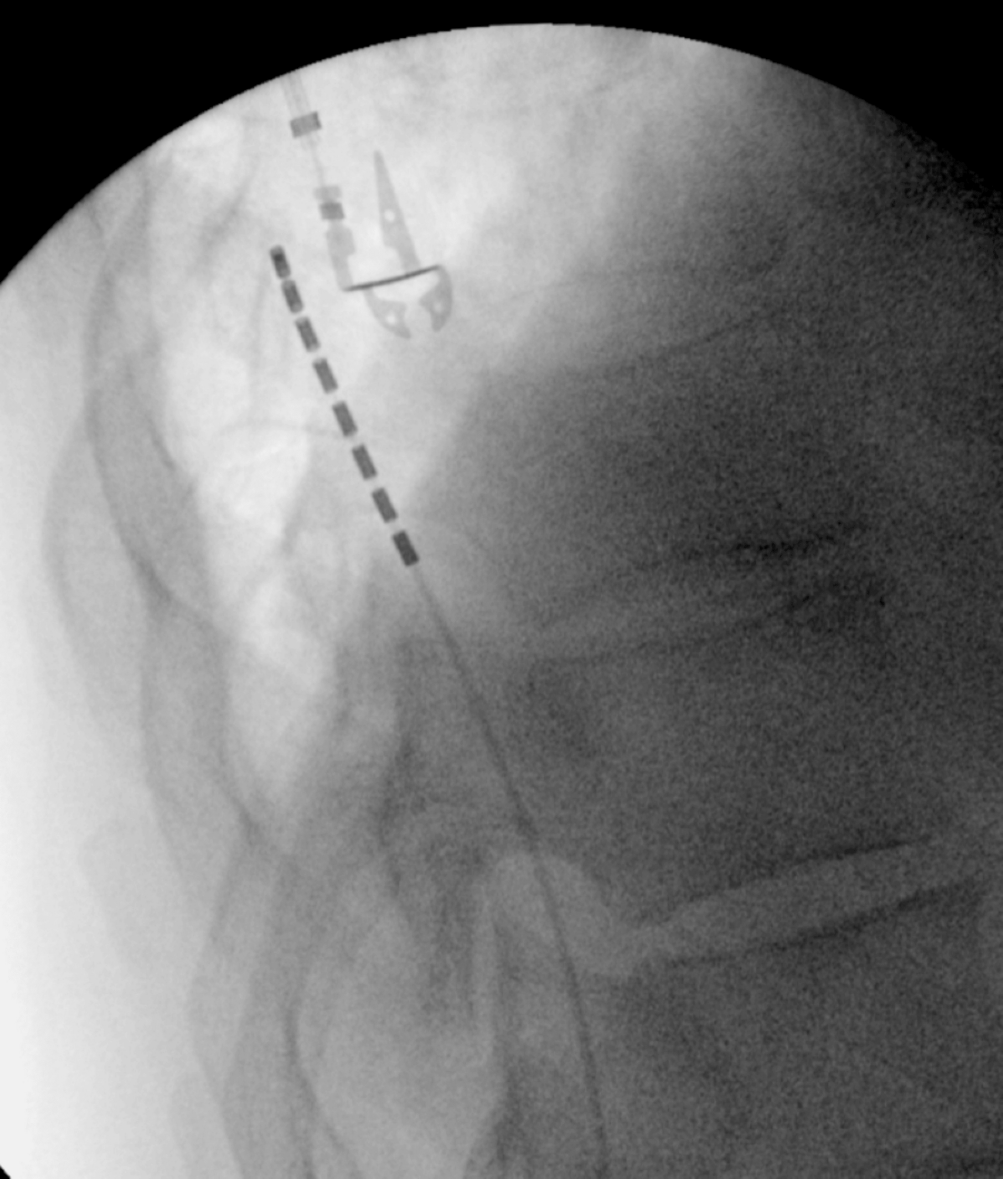
Lateral fluoroscopic image of lead revision

After passing the introducer over the lead, we guided the lead further into the epidural space using the stylet to steer the lead into the right position (Figure 7). Once a proper lead position was obtained and confirmed with fluoroscopy after the introducer was removed, a Clik anchoring device was placed over the top of the lead and anchored in the deep fascia.

A splitting device was then attached to the lead, connecting it with the battery. The battery was then placed in the pocket after copious irrigation with an antibiotic solution. The incisions of the battery pocket were closed initially with a deep closure layer and intermediate closure layer using 3-0 Vicryl, followed by a subcuticular closure with 2-0 Vicryl. After the skin closure was complete, the sites were then covered with an occlusive dressing.

The patient tolerated the procedure well, with no complications. The patient was extubated and transferred to the stretcher, where an abdominal binder was placed. He was then transferred to the PACU. The patient was discharged home and was scheduled for a 14-day follow-up appointment at our Pain Clinic.

The patient experienced a decrease in visual analog scale (VAS) score from 8 to 4 following the revision procedure. Postoperative imaging confirmed the stable position of the leads within the epidural space. Unfortunately, a few months after the revision, the patient noted a new pain in his abdomen, and reimaging of the thoracolumbar region showed a migration of the lead. The patient was referred to neurosurgery for the placement of paddle spinal cord stimulator leads.

## Discussion

The presented case underscores the intricacies and difficulties associated with percutaneous spinal cord stimulator implantation, with a particular focus on lead migration and the subsequent requirement for revision. Lead migration is acknowledged as a potential complication in SCS procedures, and endeavors to refine techniques and minimize such occurrences are crucial for optimizing patient outcomes. Several factors, such as patient movement, inadequate anchoring, and anatomical changes over time, can contribute to lead migration in SCS.

This case highlights the significance of meticulous surgical technique, thorough preoperative planning, and real-time imaging to ensure the proper placement and fixation of leads within the epidural space. Technical considerations for this case include the utilization of anchoring mechanisms and the selection of stylets for epidural space access. The challenge of scar tissue formation around the anchor complicates the revision procedure and highlights the importance of selecting and perfecting anchoring mechanisms to minimize tissue growth and facilitate future revisions.

The difficulty encountered in advancing the lead with the curved stylet raises questions about the choice of stylet and its impact on lead maneuverability. Subsequent studies may explore the efficacy of different stylets in guiding leads within the epidural space. Lead migration remains one of the most frequent causes of loss of efficacy and subsequent explantation in spinal cord stimulation systems [3,6]. Advances in anchoring technology and lead design, such as active fixation mechanisms and enhanced strain relief features, may significantly reduce the risk of migration [3,4]. By minimizing displacement, these innovations can help maintain stable stimulation coverage and ultimately lower the need for surgical revision or explantation.

A comprehensive preoperative assessment, including an evaluation of patient anatomy and risk factors for lead migration, can help select suitable candidates for percutaneous SCS and reduce the likelihood of complications [1-4,6]. The importance of exploring alternative lead designs, such as paddle leads, which may offer distinct advantages in terms of stability and coverage, is also noted in this case. The decision to convert to paddle leads should be individualized based on patient factors, including anatomy, pain distribution, and previous surgical history. Comparative studies have demonstrated the long-term efficacy and infection risks associated with paddle leads versus cylindrical SCS leads [8], but more are warranted.

## Conclusions

Migration of spinal cord stimulator leads is a potentially significant complication that may compromise the efficacy of pain management. Prompt recognition, thorough diagnostic evaluation, and surgical revision are crucial for addressing this issue. This case underscores the importance of vigilant postoperative monitoring and highlights successful strategies for the revision of migrated leads in the context of an eight-lead spinal cord stimulator. Revising cylindrical leads for percutaneous spinal cord stimulator implants requires careful consideration of technical aspects and patient-specific factors. Advances in lead design, fixation mechanisms, and a multidisciplinary approach can contribute to reducing complications, enhancing lead stability, and potentially minimizing the need for explantations or conversion to alternative lead types.
